# Functional and Aesthetic Outcomes of Chimeric vs. Single Free Flaps in Midface Reconstruction Following Tumor Resection: A Retrospective Analysis

**DOI:** 10.3390/jcm15051866

**Published:** 2026-02-28

**Authors:** Daniel Bula, Jakub Opyrchał, Łukasz Krakowczyk, Adam Maciejewski, Dominik Walczak

**Affiliations:** 1 1st Department of Oncological Surgery with Subunit of Reconstructive and Plastic Surgery, Maria Sklodowska-Curie National Research Institute of Oncology, 44-102 Gliwice, Poland; 2 2nd Department of Oncological Surgery, Maria Sklodowska-Curie National Research Institute of Oncology, 44-102 Gliwice, Poland

**Keywords:** chimeric flaps, midface reconstruction, free flaps, microvascular reconstruction

## Abstract

**Background/Objectives:** Locally advanced midface malignant tumors require extensive resection, resulting in complex defects involving bone and multiple soft tissue structures. Reconstructing these substantial defects presents a significant challenge to restore both function and aesthetics. This study aims to compare the functional and aesthetic outcomes of chimeric free flaps versus single free flaps in midface microvascular reconstructions. **Methods:** This retrospective analysis included fifty consecutive patients with Type III Cordeiro defects who underwent midface reconstruction with free tissue transfer between 2020 and 2024. The cohort included fourteen patients who received prefabricated chimeric flaps and thirty-six patients who received single free flaps. Outcomes were assessed six months postoperatively using a modified University of Washington Quality of Life Questionnaire (UW-QOL), analyzing domains including speech, chewing, sensation, appearance, pain, and social activity. Statistical analysis was performed using the Mann–Whitney U test. **Results:** In the chimeric flap group, no major flap necrosis or complications were observed. In unadjusted comparisons, the chimeric flap group showed higher transformed UW-QOL scores in several domains. Statistically significant between-group differences were observed for opening and speech (*p* = 0.004), change in appearance (*p* = 0.022), sensation (*p* = 0.011), and social activity (*p* = 0.006). Aesthetic outcomes, assessed via patient rating of appearance, were also significantly higher in unadjusted comparisons with the chimeric flap approach. Furthermore, in Type IIIa defects, titanium mesh successfully provided reliable orbital support. **Conclusions:** Chimeric free flaps represent a feasible reconstructive option in selected cases of complex maxillary and midface reconstruction. Their main advantages—providing the proper amount of specific, well-vascularized tissue and offering greater mobility of components— may be associated with more favorable functional, aesthetic, and social outcomes in unadjusted comparisons compared to reconstruction using single free flaps.

## 1. Introduction

Locally advanced malignant tumors of the middle and lower face often necessitate extensive radical resection of various structures in these regions, including the mandible, soft tissues of the oral cavity and oropharynx, maxilla, paranasal sinuses, zygoma, or orbit. Such procedures frequently involve resection of facial or neck skin and may lead to significant functional and aesthetic deficits as well as serious complications. Reconstructing post-ablative defects that encompass bone, intraoral soft tissues, and skin is a complex challenge. The choice of reconstructive method depends on multiple factors specific to each patient, such as the size and location of the defect, the patient’s age, general health status, and the patient’s expectations [1,2]. Midfacial structure consists of zygomaticomaxillary bone (ZMB), pterygomaxillary bone (PMB) and nasomaxillary buttresses (NMB). Restoration of these makes it possible to obtain optimal maxillary skeletal reconstruction [3]. Careful and precise planning of the reconstruction is critical to achieving optimal restoration. The primary goals of reconstruction are to restore skeletal support by reconstructing the mandible or maxilla, separate the nasal cavity from the oral cavity and the nasopharynx from the cranial base and to achieve facial contour, color, texture, and symmetry.

Reconstructive techniques for the middle face range from straightforward locoregional approaches to complex combinations of multiple free flaps. Advances in vascularized composite free flaps have significantly improved both functional and aesthetic outcomes. Commonly employed osteocutaneous free flaps for reconstructing these types of defects includes the iliac crest, scapula, and fibula flaps, among many others [4,5,6].

Several reconstructive strategies are available for Type III midface defects, ranging from single osseous or soft-tissue free flaps to multi-component chimeric constructs [7]. Each approach carries distinct advantages and limitations with respect to operative time, tissue versatility, and donor site morbidity. Chimeric flaps have been proposed as an option for addressing complex three-dimensional defects, although evidence demonstrating outcome differences remains limited. Therefore, an objective comparison of patient-reported results is necessary to better define their clinical role. Natural chimeric flaps (NCFs) consist of two or more distinct tissue components, each supplied by an independent vascular branch of a single main pedicle. In contrast, prefabricated chimeric flaps (PCFs) combine two or more free flaps, each with an individual pedicle. In a PCF, the distal end of the second flap’s vessel is anastomosed to the proximal end of the first flap’s vessel, resulting in a composite flap supported by a single vascular pedicle [7,8].

A key advantage of chimeric flaps over single flaps is the high mobility of their individual components, allowing for near-ideal reconstruction of diverse anatomical structures [9,10]. This study aims to present our experience and outcomes in using various natural and prefabricated chimeric flaps for reconstructing mid-face defects, with a focus on both functional and aesthetic results. Additionally, this study is trying to answer the question of whether in the case of such an anatomically complex area as the midface, the use of chimeric flaps can help achieve a better result than in the case of using single free flaps.

While several authors have reported successful applications of chimeric flaps for large midface defects, most publications emphasize surgical feasibility or complication rates rather than patient-reported outcomes [11,12]. Evidence directly comparing functional or quality-of-life parameters between chimeric and single free flap reconstructions in Type III defects remains limited, and the potential benefit of multi-component tissue design for speech and facial appearance has not been comprehensively quantified. Recent reviews highlight chimeric flaps as a promising option for complex three-dimensional defects, yet emphasize the lack of comparative outcome data supporting superiority over standard single free flap reconstruction [13,14,15,16]. Therefore, this study seeks to explore whether the theoretical advantages of multi-component design translate into measurable improvements in postoperative QOL.

The aim of this study was to compare functional, aesthetic, and quality-of-life outcomes in patients undergoing midface reconstruction with chimeric free flaps versus single free flaps following advanced oncologic resections. Given the anatomical complexity of Type III Cordeiro defects, we sought to evaluate whether the multi-component design of chimeric flaps could be associated with more favorable outcomes in speech, chewing/swallowing, sensation, appearance, and social functioning.

## 2. Methods

### 2.1. Patient Selection, Defect Classification, and Reconstructive Strategy

Inclusion criteria for the examined patient group included histologically confirmed malignant tumor involving the midface requiring ablative resection classified as Type III according to Cordeiro; reconstruction using microvascular free flaps performed between January 2020 and December 2024; availability of postoperative radiotherapy and minimum 6-month clinical and functional follow-up; and completed UW-QOL questionnaire at 6 months. To achieve a clear comparison, we excluded patients with incomplete follow-up or missing QOL data; palliative resections without intent for functional restoration; and prior reconstruction of the midface with free tissue transfer.

Flap selection was determined by defect complexity, required tissue components, surgeon assessment, and patient-specific reconstructive goals. Single flaps were used for cases in which bone or soft-tissue volume could be achieved with a single donor site, whereas chimeric flaps were preferentially selected when multiple independently vascularized components were necessary, particularly in extensive composite resections involving simultaneous palate/nasal lining reconstruction with bony structure replacement. Surgeons did not randomize patients, and flap choice reflects clinical judgment rather than standardized allocation.

We recognize that the chimeric cohort was younger on average and presented with more complex defects, which may influence postoperative recovery and functional outcomes. Due to sample size limitations, multivariable adjustment was not feasible; therefore, confounding cannot be completely excluded. This limitation has been explicitly acknowledged in the Discussion.

To describe the extent of midfacial resections, the classification system with four defect types proposed by Cordeiro was applied [7]. In this classification, Type I and II defects include limited or subtotal maxillectomy, Type III include resection of all six walls of the maxilla, and can be divided into Type IIIa, where orbital contents are present, and Type IIIb, where orbital contents are exenterated. Type IV includes orbitomaxillectomy with preservation of the palate. The principles of reconstruction were based on size and extension of the postresective defect, and on maxillary buttresses conception.

All analyzed cases where chimeric flaps were prefabricated refers to Type IIIa and IIIb. In chimeric flap cases, MRI and 3D-CT imaging were used to define preoperative plan of resection and reconstruction.

**Figure 1:**
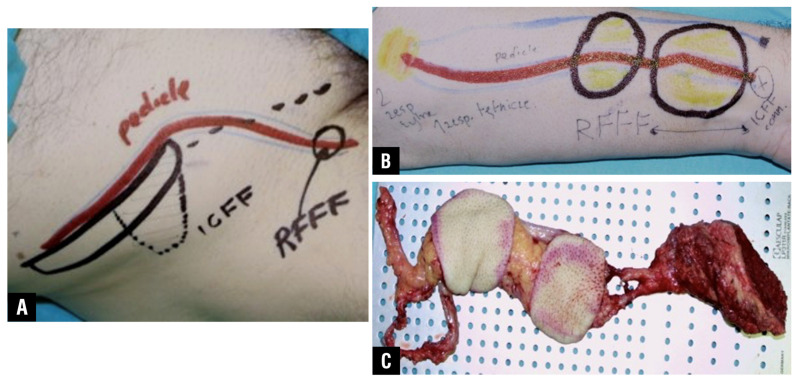
Design and intraoperative appearance of a prefabricated chimeric flap based on a radial forearm free flap (RFFF) combined with an iliac crest free flap (ICFF), demonstrating independently vascularized components used for reconstruction of bony support and soft-tissue lining. (**A**) Preoperative skin marking and schematic design of the chimeric flap, illustrating the planned pedicle course, ICFF bone fragment, and the vascular arrangement allowing incorporation of the RFFF. (**B**) Diagrammatic intraoperative planning of a double-island RFFF configuration, showing two separate skin paddles supplied by a common vascular pedicle, intended for sequential reconstruction of the palate and nasal cavity lining. (**C**) Harvested prefabricated chimeric flap demonstrating the assembled construct, with the ICFF vascularized segment anastomosed in an end-to-end fashion to the distal radial artery and vein. The cephalic vein is included as an auxiliary venous outflow to enhance drainage and reduce the risk of venous congestion.

**Figure 2:**
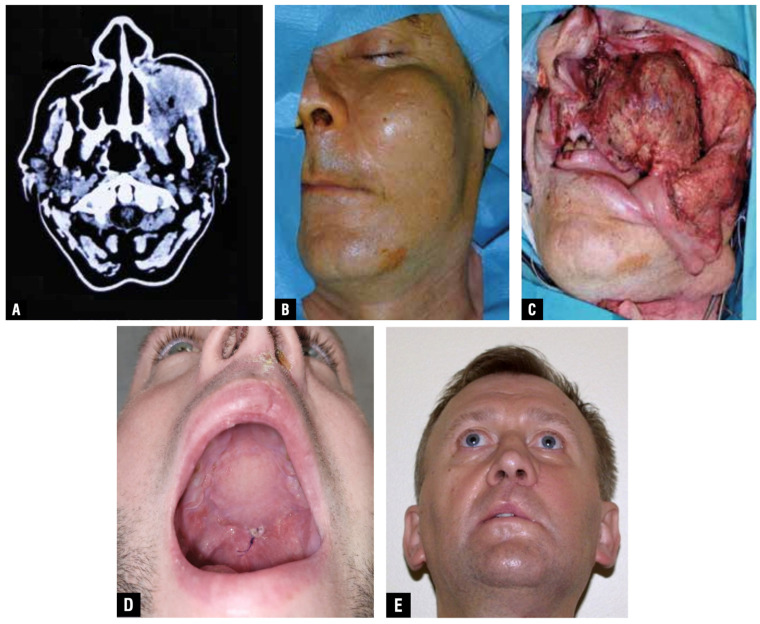
Representative imaging of Case 7 undergoing reconstruction of a Type III midfacial defect with a chimeric free flap (double-island radial forearm free flap combined with a fibula free flap). (**A**) Preoperative axial computed tomography demonstrating an advanced unilateral midface defect involving the maxillary region and adjacent structures, consistent with a Type III defect. (**B**) Preoperative semi-lateral clinical view showing external facial contour deformity and loss of midfacial projection. (**C**) Intraoperative photograph following ablative resection, illustrating the extent of the composite midface defect immediately after inset of the chimeric flap. (**D**) Six-month postoperative intraoral view demonstrating stable integration of the soft-tissue component of the reconstruction with adequate palatal lining and mucosal healing. (**E**) Six-month postoperative frontal–inferior clinical view showing restoration of midfacial contour and projection, with satisfactory facial symmetry and soft-tissue stability.

**Figure 3:**
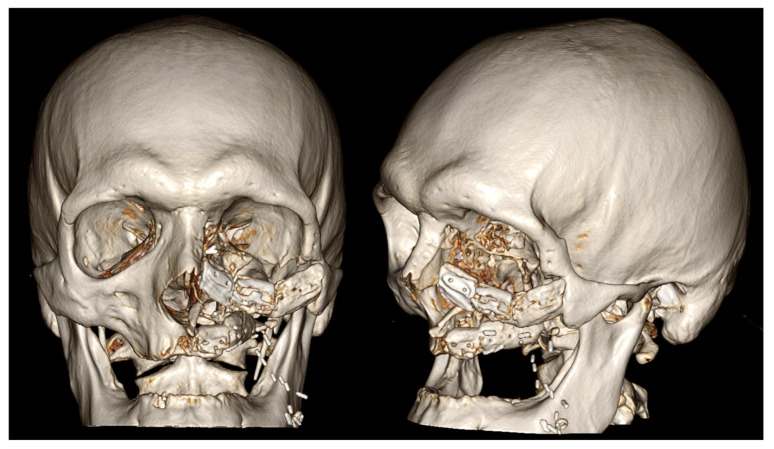
Postoperative three-dimensional computed tomography (3D-CT) illustrating Case 7, in which a chimeric reconstruction combining a fibula free flap (FFF) and a double-island radial forearm free flap (RFFF) was performed. The fibula free flap was used to reconstruct the alveolar process and the zygomatic process of the maxilla, restoring the osseous framework of the midface.

In 12 cases with Type IIIa defects, six walls of the maxilla were resected together with surrounding tissues, but the orbit was preserved. In these patients, double skin paddle radial forearm free flap was anastomosed to the iliac crest free flap (5 cases) (Figure 1), or fibula osseous free flap (7 cases) (Figure 2). The proximal vessels of vascularized bone were micro-anastomosed to distant end of radial artery and vein; therefore, the main pedicle of sequential chimera was created by proximal radial vessels. Double radial skin islands reconstructed both nasal cavity and palate. In cases of chimera with fibula flap, bone osteotomies were performed to reconstruct PMB and ZMB with help of virtual planning and 3D-printed cutting guides (Figure 3). The inferior orbital wall was reconstructed with titanium mesh. In five cases where iliac crest was used, the bone with no osteotomies was used to recreate both PMB and ZMB; therefore, the bone reconstructed the alveolar ridge and lower orbital wall.

Two remaining cases represented Type IIIb defects, where extended maxillectomy was combined with orbital exenteration. In both cases, combination of radial forearm osteocutaneous free flap with vertical rectus abdominis free flaps was applied. Radial skin islands made it possible to recreate both nasal cavity and palate. The osseous part of the flap, with two osteotomies, reconstructed PMBs. To achieve bulkiness, the maxillary and orbital space was filled with the rectus abdominis muscle.

In both subtypes of defects, the aim of soft tissue reconstruction was to recreate the nasal cavity (first radial skin island), and to separate the internal nasal cavity from oral cavity (second radial skin paddle). In addition, in Type IIIb, the rectus abdominis muscle was used to fill the orbitomaxillary space. The process of creating sequential chimeric flaps involved first harvesting the distal flap, followed by preparing the proximal flap, and finally connecting the pedicle of the distal flap to the distal section of the proximal flap.

In Type IIIa the inferior orbital wall was recreated with the titanium mesh, before flaps detachment. Afterwards, the complex was detached and brought to the defect. In all cases, arterial anastomoses were performed in an end-to-end fashion between the facial artery and radial artery, and a venous drainage was established with the internal jugular vein system (in two cases double anastomoses were performed). Due to the advanced stage of the cancer and the treatment guidelines in our center, all patients received postoperative radiotherapy with the total dose from 62 to 68 Gy.

All patients received standardized postoperative pharmacological thromboprophylaxis consisting of low-molecular-weight heparin (LMWH) and acetylsalicylic acid (ASA, 75 mg daily) for 14 days following surgery. Postoperative flap monitoring was performed using a combined protocol based on regular clinical assessment and adjunctive handheld Doppler ultrasonography. Clinical evaluation included assessment of flap color, capillary refill, temperature, and tissue turgor, while Doppler examination was used to confirm vascular flow within the pedicle during the monitoring period.

### 2.2. Evaluation of Outcomes

The University of Washington Quality of Life Questionnaire (UW-QOL, version 4) was used as the basis for postoperative quality-of-life assessment [17]. For the purposes of this study, we applied a modified domain subset tailored to midface oncologic reconstruction. From the original instrument, six domains most relevant to functional recovery after maxillectomy were analyzed: pain, chewing/swallowing, speech, oral sensation, appearance, and social activity. Items not directly associated with midface function (e.g., shoulder function) were excluded to reduce patient assessment burden and focus on outcomes that may differ between chimeric and single free flap reconstructions. No wording of individual questions was altered; only the number of domains included in the final scoring set was adjusted. This approach follows previously reported use of domain-specific UW-QOL extractions in head and neck reconstruction studies, where selected subscales have been used to evaluate targeted functional recovery. The modification does not replace the validated questionnaire but uses specific items within the validated framework to measure outcomes relevant to this patient population. For clarity, the 0–100 transformed scores are used only to make domain scores easier to compare. They are not a validated UW-QOL composite score and should not be treated as a quality-of-life benchmark. The implications of using a subset of domains are acknowledged as a study limitation in the Discussion. All quality-of-life assessments were completed at a standardized six-month postoperative time point and prior to the occurrence of any documented local or nodal recurrence.

This assessment evaluated the impact of reconstruction on patients’ quality of life (QOL) relative to the microvascular free flap technique employed. Six domains were analyzed: pain, chewing and swallowing, sensation, speech, appearance, and social activity. To enhance transparency and illustrate the variability of outcomes within the chimeric cohort, we presents raw, patient-level domain scores for this subgroup in Section 3. Individual scoring was shown for chimeric cases because of the smaller sample size (n = 14) and the exploratory nature of evaluating multi-component flap performance. For the single free flap group (n = 36), raw scores were not displayed individually to maintain table readability and avoid excessively large data formatting.

Raw domain scores (RS) for each UW-QOL item were converted to a standardized 0–100 scale to allow comparison between groups. Functional scales (chewing/swallowing, speech, sensation) were coded so that higher values indicated better function, whereas symptom-related scales (pain, appearance change) were oriented so that higher scores reflected greater symptom severity. Standardized scores were calculated using the following:

- **Functional scales:** $AverageScore=\left[1-\frac{\left(RS-1\right)}{range}\right]\times 100$
- **Symptom scales:** $AverageScore=\left[\frac{\left(RS-1\right)}{range}\right]\times 100$

Thus, lower raw values in functional domains yield higher standardized scores, while higher raw symptom scores increase standardized values. This conversion method allows direct numerical comparison across outcomes with differing raw ranges.

A detailed conversion table demonstrating step-by-step score transformation for one representative patient has been included as Supplementary Table S1 to support reproducibility. Outcomes for the 14 patients reconstructed with chimeric flaps were compared to QOL scores of the remaining 36 patients who underwent single free flap reconstruction. Average QOL scores for chimeric flap cases were recalculated as transformed functional scale (FS) and symptom scale (SS) scores and compared to those of single free flap reconstructions.

Continuous outcome variables were compared between chimeric and single free flap groups using the Mann–Whitney U test due to non-parametric distribution of QOL scores and the limited sample size. Descriptive statistics are presented as mean values. As mentioned previously, six UW-QOL domains were analyzed, selected a priori as most relevant to function and aesthetics after midface reconstruction. Because this retrospective study was designed as an exploratory, hypothesis-generating comparison and the chimeric cohort was small, no formal adjustment for multiple testing was applied; therefore, domain-level *p*-values are reported as unadjusted and should be interpreted as exploratory rather than confirmatory. Given the retrospective nature of the cohort and the relatively small number of chimeric reconstructions, multivariable modelling was not performed; therefore, the results represent unadjusted comparisons and may be influenced by confounding factors such as tumor extent, defect complexity, and individual baseline function. All statistical analyses therefore represent unadjusted, exploratory comparisons, and observed associations cannot be interpreted as causal or technique-specific effects. This limitation is addressed in the Discussion. All consecutive patients operated on during the study period with available six-month follow-up were included in the analysis. Patients with incomplete questionnaires or lacking postoperative assessment were excluded from the final comparison dataset. No imputation was applied for missing QOL responses to avoid artificially altering outcome distribution. Postoperative complications were recorded prospectively and analyzed descriptively; however, they were not incorporated as covariates in comparative statistics. Follow-up variability was minimized by evaluating functional outcomes at a uniform six-month postoperative time point. All statistical analyses were performed using the R environment 4.2.0. (R Foundation for Statistical Computing, Vienna, Austria). Statistical significance was set at *p* < 0.05.

## 3. Results

### 3.1. Patients’ Characteristics

Among fifty consecutive patients presenting with Type III Cordeiro defects who underwent middle face reconstruction with free tissue transfer, thirty-six received a single free flap reconstruction. A soft-tissue flap was selected in approximately half of these cases, while eighteen patients underwent reconstruction using osteocutaneous free flaps (Table 1). The mean age of patients in the single free flap cohort was 65.2 years (SD 8.9), whereas the mean age in the chimeric flap group was 57.7 years (SD 8.4). The analyzed cohort included fourteen patients in whom prefabricated chimeric flaps were utilized (Table 2). Baseline demographic and tumor-related characteristics differed between reconstructive cohorts. Patients reconstructed with chimeric free flaps were younger on average compared with those reconstructed using single free flaps. A history of tobacco use and high alcohol consumption was common in both reconstructive groups, reflecting the typical risk profile of patients presenting with advanced midface malignancies. In the chimeric flap group, Cordeiro Type IIIa defects predominated (12/14; 85.7%), with Type IIIb defects accounting for 2/14 (14.3%), whereas in the single free flap group, Type IIIa defects accounted for 23/36 (63.9%) and Type IIIb defects for 13/36 (36.1%). Tumor histology was predominantly squamous cell carcinoma in both groups, with comparable distributions of T and N categories. All patients were treated with curative intent and underwent postoperative radiotherapy according to institutional protocols. Because of the retrospective design and non-randomized flap selection, baseline differences reflect clinical indication rather than standardized allocation. Surgical procedures were performed by the same reconstructive team between 2020 and 2024. Table 3 is provided to detail the technical composition and variability of chimeric reconstructions and is not intended as a direct comparison with the single free flap cohort. A total of fifty patients underwent oncologic resection with reconstruction for Type III midface defects. Fourteen patients (28%) were reconstructed with chimeric free flaps and thirty-six (72%) with single free flaps. All resections achieved R0 margins based on intraoperative frozen evaluation and final histopathology. Baseline patient and flap characteristics are summarized in Table 1, Table 2 and Table 3. Reconstructions performed with chimeric flaps were associated with a longer operative duration compared with single free flap procedures (437.4 ± 54.9 min vs. 314.4 ± 40.5 min; *p* < 0.001). Mean ICU stay was comparable between the chimeric flap and single free flap groups (2.6 ± 1.3 vs. 3.0 ± 1.5 days; *p* = 0.296), as was mean total hospital stay (11.1 ± 3.0 vs. 9.9 ± 3.1 days; *p* = 0.186), with no statistically significant differences observed. No major donor-site complications were observed in either study group. Donor sites healed without clinically significant wound complications, functional impairment, or aesthetic concerns requiring additional intervention. This included patients undergoing chimeric reconstructions involving multiple donor sites, in whom no donor-site–related adverse events were recorded during the follow-up period.

### 3.2. Postoperative Complications

Total postoperative complications for both groups were observed in 10 patients (20%). In the chimeric group, complications included prolonged wound healing in 2/14 (14.3%) and partial skin island ischemia requiring revision in 1/14 (7.1%). In the single free flap cohort (n = 36), complications included partial necrosis in one patient (2.8%), oro-cutaneous fistula formation in two patients (5.6%), and delayed wound healing in three patients (8.3%). Due to postoperative cardiorespiratory failure, one patient (2.8%) required high-dose pressor therapy, which led to complete free flap necrosis (ALTF). This patient underwent repeat reconstruction with a volumetric RAM flap. In the chimeric cohort (n = 14), flap survival was 100% with no complete flap loss. Overall postoperative complications occurred in 3 of 14 patients in the chimeric flap group (21.4%) and 7 of 36 patients in the single free-flap group (19.4%), indicating a comparable complication burden between groups despite 100% flap survival in the chimeric cohort. In Type IIIa defects reconstructed with inferior orbital wall support using titanium mesh, no cases of diplopia, enophthalmos, or mesh exposure were observed during follow-up. These findings are descriptive and should be interpreted cautiously, as no formal comparison or quantitative orbital-volume assessment was performed. While the absence of orbital complications is encouraging, it should be viewed as hypothesis-generating rather than evidence of superiority over alternative reconstructive methods. Among the chimeric flap group, no major complications were observed. In patient No. 6, wound healing was prolonged, but surgical intervention was not required. In one patient (No. 10) ischemia of the radial flap skin island was reported, which was caused by thrombosis of the anastomosis between the free radial and fibula flap. The patient required revision 5 h after the initial procedure. The anastomosis was excised and re-performed. This was possible due to the long pedicle of the radial flap. In the further postoperative period, no additional complications were observed.

### 3.3. Raw vs. Standardized Scoring

Table 4 presents raw UW-QOL scores, whereas Table 5 displays standardized 0–100 transformed values. Raw scores reflect direct patient responses (lower raw values indicating better function for functional domains), while transformed scores normalize different domain scales for cross-domain comparison. Transformed QOL scores are presented on a standardized 0–100 scale to facilitate comparison across domains with different raw score ranges. Therefore, differences in transformed scores should be read as exploratory, domain-level comparisons of re-scaled answers, not as changes in a validated overall quality-of-life score. In unadjusted, exploratory domain-level comparisons, the chimeric flap group demonstrated higher transformed scores in several domains; the smallest *p*-values were observed for opening and speech (*p* = 0.004), change in appearance (*p* = 0.022), sensation (*p* = 0.011), and social activity (*p* = 0.006). In unadjusted comparisons, the chimeric flap group had higher transformed scores in several key domains, notably speech, chewing/swallowing, sensation, appearance, and social integration. Although formal effect size estimates with confidence intervals could not be calculated due to sample size limitations and non-normal score distributions, the direction of differences was consistent across several functional domains. Furthermore, these transformed values do not constitute a validated composite quality-of-life index but represent exploratory, normalized domain-level scores derived from selected UW-QOL items to facilitate between-group comparison. They should be interpreted as associations rather than technique-related effects, considering domain-specific score orientation and transformation, as well as baseline differences between cohorts and indication-driven flap selection, and should not be viewed as uniform improvements across all outcome scales.

### 3.4. Descriptive Clinical Outcomes

Beyond statistical significance, unadjusted between-group differences were observed in clinically relevant descriptors: 80% (11/14) of chimeric cases achieved normal speech vs. 39% (14/36) in single free flap patients. Six out of fourteen (43%) resumed unrestricted diet intake vs. 9/36 (25%) in the single free flap group. Median social activity score in the chimeric group was nearly double that of single free flap reconstructions (75 vs. 40). Aesthetic perception differed by approximately 20 points. While differences in transformed scores showed consistent directionality favoring the chimeric flap group in selected domains, no formal effect size estimates or confidence intervals were calculated; therefore, these findings should be regarded as exploratory and descriptive rather than indicative of measurable effect strength.

### 3.5. Salvage Treatment

During follow-up, six local recurrences (12%) and one nodal recurrence (2%) were identified among the 50 patients. As part of the institutional protocol, all patients received postoperative radiotherapy (62–68 Gy) following surgical reconstruction, independent of flap type. None of the patients underwent routine postoperative concurrent chemoradiotherapy.

Management of recurrence consisted of salvage systemic chemotherapy and resection, rather than adjuvant therapy in the classical postoperative sense. The seven patients who had recurrence initiated salvage treatment after the six-month QOL assessment window; therefore, subsequent systemic therapy is not expected to have influenced the outcome measures presented in this study. Long-term functional effects of salvage therapy were not evaluated and fall outside the scope of the current analysis.

## 4. Discussion

Historically, palliative treatment was the only option for most patients with advanced cancer of the middle face. However, advancements in reconstructive surgery, with or without adjuvant therapy, have provided the possibility of radical treatment with improved prognostic outcomes. For many years, muscular flaps, such as latissimus dorsi and rectus abdominis, were widely used in reconstruction [18,19]. These techniques allowed for the transfer of large volumes of muscle and skin to address post-resection defects [20]. However, the functional and aesthetic outcomes were often suboptimal. In addition to pedicled flaps, the traditional approach for middle face restoration frequently relied on prosthetic obturators. This nonsurgical method was effective in closing oronasal communications but was prone to failure, particularly in patients experiencing oronasal leakage or reflux into the nasal cavity. In 1986, Swartz et al. introduced the scapular osteocutaneous free flap, enabling simultaneous reconstruction of the orbital wall and alveolar ridge [21]. Subsequently, Sadove and Powell utilized free fibula osteocutaneous flaps for reconstructing maxillary and mandibular defects [22]. Despite these advancements, the authors found it challenging to simultaneously reconstruct both bony and soft tissue structures using a single vascularized osseous flap. The concept of chimeric flaps was introduced by Koshima et al. in the early 1990s [5]. Their research demonstrated the superiority of sequential, multi-component flaps over earlier reconstruction methods. Based on the chimeric flap principle, several vascularized graft options have been developed for addressing massive, extended midface defects. Chimeric flaps consist of multiple tissue components, each supplied by an independent vascular pedicle. Hallock described a combination of rectus femoris and anterolateral thigh flaps, both vascularized by branches of the lateral circumflex femoral artery [10]. Other examples include the LD/serratus anterior muscle flaps and scapular/parascapular flaps. Several reports suggest that these techniques may offer improved cosmetic and functional outcomes compared with conventional microvascular approaches, particularly in complex three-dimensional defects. Some authors advocate combining soft tissue flaps with prostheses, a technique where the flap restores internal nose structures and facial contour while the prosthesis separates the nasal and oral cavities, reconstructs the alveolar ridge, and closes the defect [23]. However, this approach requires close collaboration between reconstructive surgeons and prosthetic specialists. Due to the lack of in-house palate prosthetics and dental rehabilitation services at the authors’ center, the goal of single-stage reconstruction was to restore both osseous and soft tissue structures. Based on experience, the authors found that the optimal results were achieved by using reconstructive materials that matched the missing tissue types. The authors introduced various chimeric flaps, emphasizing the importance of precise bone shaping and fixation, particularly in the posterior maxilla, to achieve the best functional and aesthetic outcomes. In Type IIIa defects according to the Cordeiro classification, where maxillectomy includes the orbital floor but spares the eyeball, the orbital contents can prolapse into the defect [11]. Various techniques have been described for orbital support reconstruction, but few studies have exclusively addressed maxillectomy defects with orbit preservation. Cordeiro suggested that nonvascularized bone grafts can effectively reconstruct the orbital floor. In the study group, orbital support was restored using titanium mesh, placed between the soft tissue component of the flap below and the orbit above. The mesh successfully maintained the eyeball in its anatomical position in all cases. In all of the analyzed cases (14), good postoperative functionality was achieved, including satisfactory outcomes for chewing, swallowing, mouth opening, and speech. Although titanium mesh demonstrated stable support without diplopia or enophthalmos in our Type IIIa cases, these results represent observational outcomes within a limited cohort. Quantitative measurements of orbital projection or comparative control data were not available; therefore, these findings should be regarded as preliminary and require prospective validation. These results underline the importance of tailored, multi-component reconstructive approaches in achieving optimal functional and aesthetic outcomes for middle face defects.

Historically, single free flaps have enabled reliable closure of midface oncologic defects; however, they frequently provide limited ability to simultaneously reconstruct bone, mucosa, and external skin with independent tissue orientation. Several authors have demonstrated that reconstructive strategies allowing for three-dimensional tissue tailoring, particularly chimeric flaps, improve restoration of oral sealing, palatal competence, and midfacial projection, which collectively influence speech, diet, and social reintegration [5,6,7,12,13,14,15]. Chimeric concepts permit vascularly independent components to be positioned with greater freedom, improving contour restoration and reducing the need for secondary revisions. Because patient-reported outcomes strongly reflect function and aesthetic perception, it is reasonable to hypothesize that superior anatomic reconstruction could translate into better quality-of-life (QOL) scores, especially in domains related to speech, appearance, and social activity. Despite these theoretical advantages, comparative clinical data remain limited, creating a need to evaluate whether chimeric flaps effectively improve patient-centered outcomes in advanced midface resections.

Based on our findings, reconstruction with chimeric flaps may represent a feasible option in selected patients undergoing advanced midface resections, particularly when defects involve multiple anatomical structures. In this study, functional outcomes such as speech and social activity tended to be more favorable in the group receiving chimeric flaps, with some statistically significant differences observed. Aesthetic outcomes also appeared somewhat improved (Table 5). These effects may be related to the versatility of chimeric flaps, which can combine appropriately sized bone segments (e.g., fibula or iliac crest free flaps) with soft tissue components (e.g., radial forearm free flap) [12].

In many cases, when harvesting a bone flap, it turns out that the perforator supplying the skin island is too small to take enough tissue. The chimeric sequential flap eliminates this possibility. An additional aspect that should be mentioned is the mobility of the tissue elements. Since practically every element is taken on its vascular pedicle, it is possible to adjust this tissue complex to the postresective defect. The selection of the group of patients included in this study is not accidental. The study group includes patients suffering from midface cancer who require the most sophisticated reconstructions because of the neoplasm, and due to their characteristic anatomical structure, these most often involve many types of tissues and structures. As presented in Section 2, in the case of chimeric flaps, each element played a key role in maintaining the bone scaffold and isolating the oral cavity from the nasal cavity.

However, in addition to many positive aspects, chimeric flaps present many challenges, both to the surgeon and to the patient. Due to the level of complexity, only an experienced microsurgeon can perform the reconstructions described above. Additionally, especially in today’s times, the financial aspect cannot be omitted. Procedures utilizing chimeric flaps take significantly longer operating times. This affects both the patient (they are subjected to longer anesthesia) and the work of the operating theater in the hospital [13]. In the case of possible complications, it seems that single free flaps are a much better choice than chimeric flaps. In the case of the latter, more microanastomoses are performed on the patient, so there is a higher risk of thrombotic/ischemic complications. In the present cohort, chimeric flap reconstruction was not associated with an increased rate of major flap loss, and only one case required early revision of an inter-flap anastomosis, which was successfully salvaged. Overall complication rates were comparable between reconstructive strategies, although this study was not powered to detect small differences in uncommon adverse events. Additionally, there are two donor sites for free flaps, so theoretically the risk of complications doubles. However, in a selected group of patients, chimeric flaps may be the only chance to achieve the acceptable functional and aesthetic effect. These are exceptional patients whose cancer is so advanced that reconstruction with standard single flaps will not allow for satisfactory results, and at the same time they are healthy enough to survive a prolonged procedure and a theoretically higher risk of complications. Based on the present findings, the use of modern reconstructive techniques, including chimeric flaps, may be considered in selected patients with highly complex midface defects, particularly when standard single free flap reconstruction is unlikely to provide adequate structural or functional restoration. It is worth adding that chimeric flaps are also successfully used in the reconstruction of advanced lower facial tumors [14].

Only a limited number of studies have attempted to evaluate postoperative function or patient satisfaction after chimeric midface reconstruction, and direct comparison with single free flaps is rarely reported. Our findings provide hypothesis-generating evidence suggesting a potential functional and psychosocial advantage of chimeric flaps in high-defect complexity settings.

Currently, there is a growing number of modifications to well-known surgical techniques, primarily based on the presentation of complex tissue using commonly used flaps [15]. However, it is important to remember that treatment of advanced midface cancers begins well before the procedure itself [24]. Preoperative planning and selection of high-risk patients are crucial in these cases [25,26,27]. Furthermore, increasingly sophisticated treatment algorithms have been emerging for a long time [28,29,30]. However, in the authors’ opinion, patients requiring midface reconstruction require an individualized, often unconventional, approach. Patients requiring midface reconstruction may benefit from an individualized reconstructive strategy tailored to defect complexity and patient-specific factors, which can contribute to acceptable aesthetic and functional outcomes. Chimeric flaps seem to be an ideal aspect to expand the armamentarium of the reconstructive surgeon. Although recurrence management did not affect our primary six-month QOL outcomes, salvage systemic therapy administered thereafter may influence longer-term function and psychosocial recovery, and future longitudinal studies should incorporate recurrence-adjusted analyses.

When interpreted in the context of the existing literature, our findings are broadly consistent with previously reported advantages of chimeric or multi-component reconstruction in complex head and neck defects. Ettyreddy et al. reported that chimeric free flaps demonstrate complication rates comparable to conventional free flap strategies while offering greater flexibility for three-dimensional reconstruction, particularly in composite defects involving bone and soft tissue [12]. More recent comparative analyses by Wang et al. showed that chimeric flaps may reduce the need for multiple simultaneous free flaps without increasing overall morbidity, suggesting potential functional advantages in carefully selected patients [13]. Similarly, a recent systematic review and meta-analysis by Punjabi et al. found no significant increase in flap failure or major complications with chimeric reconstructions compared with multiple free flaps, while emphasizing the theoretical benefit of improved tissue positioning and contour restoration [14].

Unlike these studies, which primarily focused on surgical feasibility and complication profiles, the present analysis emphasizes patient-reported functional and psychosocial outcomes following midface reconstruction. Although direct comparison is limited by differences in defect location and outcome metrics, the observed association between chimeric reconstruction and higher QOL scores in speech, appearance, and social activity is conceptually aligned with the functional rationale proposed in prior studies. Nevertheless, given the retrospective design and lack of adjustment for confounding, these findings should be interpreted as complementary to, rather than confirmatory of, existing evidence.

This study has several limitations that should be considered when interpreting the results. The retrospective and non-randomized design introduces potential selection bias, and although the two reconstructive groups were operated on during the same period by the same surgical team, unmeasured confounders such as tumor extent, defect complexity, or patient-specific functional reserve may have influenced outcomes. The sample size, particularly within the chimeric flap cohort, was relatively small, which limits statistical power and may affect the ability to detect more subtle differences between techniques. Flap selection was not randomized but based on clinical judgment, defect morphology, and anticipated reconstructive requirements. Consequently, selection bias and confounding by indication may have influenced outcomes, and the functional differences observed in the chimeric group should be interpreted as associative rather than causal. Larger prospective studies with matched cohorts or multivariable adjustment will be required to better isolate the effect of flap type from patient- and tumor-related factors. All patients received postoperative radiotherapy, which may influence recovery of speech, sensation, and oral intake regardless of reconstruction type. As described in Section 2, quality-of-life outcomes were derived from a modified subset of the UW-QOL instrument focusing on domains most relevant to midface reconstruction. Although this approach preserved the original item wording and mapped to validated UW-QOL constructs, the non-standard domain aggregation and score transformation may influence interpretation of results. Therefore, the 0–100 values should not be compared to published UW-QOL reference or normative scores; they are only for comparisons within our cohort. Differences in domain directionality and weighting could potentially overestimate or underestimate between-group differences. Therefore, the observed QOL associations should be interpreted cautiously and regarded as exploratory. Prospective studies employing fully standardized patient-reported outcome measures are required to validate these findings. Finally, functional evaluation was conducted at six months postoperatively; longer follow-up may reveal additional changes related to adaptation, rehabilitation, or delayed aesthetic refinement. We did not calculate formal effect size estimates or confidence intervals, and statistical inference was based on non-parametric group comparisons; therefore, *p*-values should be interpreted cautiously and not as definitive measures of effect magnitude. Prospective studies with larger cohorts, unmodified QOL instruments, and multi-institutional comparison would be valuable to further validate these findings and define optimal patient selection for chimeric reconstruction.

## 5. Conclusions

Based on our institutional experience, chimeric flaps appear to be a feasible reconstructive option in selected cases of complex maxillary defects, providing access to well-vascularized, tissue-specific components with favorable mobility for three-dimensional restoration. In our retrospective cohort, we observed more favorable patient-reported outcomes, particularly within functional and psychosocial outcome domains, including speech, superficial sensation, aesthetic appearance, and social activity, but because quality-of-life outcomes were derived from normalized, non-standardized domain-level transformations rather than a validated composite score, the observed numerical differences should be interpreted cautiously and considered hypothesis-generating. While this technique may offer advantages with acceptable donor-site morbidity, further prospective, controlled studies are necessary to confirm these associations and better define patient selection criteria. These findings are hypothesis-generating and primarily inform feasibility and patient selection; any conclusions regarding technique-level advantage should be interpreted cautiously.

## Figures and Tables

**Table 1:** Summary characteristics of patients reconstructed with a single free flap, aggregated by flap type. Values are presented as means. Defect area represents the surface area calculated from the two largest recorded defect dimensions (cm^2^) and is used for descriptive comparison only. In one patient reconstructed with an anterolateral thigh flap, complete flap necrosis occurred and was managed with secondary reconstruction using a rectus abdominis muscle flap.

Flap Type (n)	Mean Age (Years)	Mean Defect Area (cm^2^)	Complications/Comments
Anterolateral thigh flap (ALTF) (n = 10)	68.5	72	Delayed wound healing (n = 2); Complete flap necrosis requiring secondary RAM reconstruction (n = 1)
Iliac crest free flap (ICFF) (n = 9)	63.7	36	None
Fibula free flap (FFF) (n = 6)	67.8	46	Delayed wound healing (n = 1)
Rectus abdominis muscle flap (RAM) (n = 5)	65.2	80	Delayed wound healing (n = 1); orocutaneous fistula (n = 2)
Radial forearm free flap (RFFF) (n = 3)	67.0	34	None
Radial forearm bone free flap (bRFFF) (n = 3)	59.7	38	Partial flap necrosis (n = 1)

**Table 2:** Brief characteristics of prefabricated chimeric flaps group. SCC—Squamous Cell Carcinoma; TNM—TNM Classification of Malignant Tumors.

No.	Sex	Age	Tumor Type	TNM Classification
1	M	65	SCC	T4 N0 M0
2	F	52	Adenocarcinoma	T4 N0 M0
3	M	50	SCC	T4 N1 M0
4	M	63	Adenoid cystic carcinoma	T4 N0 M0
5	F	48	SCC	T4 N0 M0
6	M	54	SCC	T4 N0 M0
7	M	56	SCC	T4 N1 M0
8	M	48	Sarcoma	T4 N1 M0
9	M	62	SCC	T3 N0 M0
10	F	71	SCC	T4 N0 M0
11	F	69	SCC	T3 N1 M0
12	M	47	Sarcoma	T4 N0 M0
13	F	65	Adenoid cystic carcinoma	T3 N0 M0
14	M	58	SCC	T4 N0 M0

**Table 3:** Case-level characteristics of patients reconstructed with chimeric free flaps, including defect classification, flap composition, and postoperative complications. RFFF—Radial Forearm Free Flap, ICFF—Iliac Crest Free Flap, FFF—Fibula Free Flap, VRAM—Vertical Rectus Abdominis Muscle Flap.

No.	Soft Tissue Defect	Resection	Type (Classification by Cordeiro)	Type of Chimeric Flap	Postoperative Complications
1	7 × 4 × 4 cm	Maxillectomy + alveolar ridge + hard palate + medial nasal wall	IIIa	Double island RFFF + ICFF	None
2	7 × 8 × 6 cm	Maxillectomy + alveolar ridge + hard palate + medial nasal wall + buccal soft tissues	IIIa	Double island RFFF + ICFF	None
3	7 × 6 × 5 cm	Maxillectomy + alveolar ridge + hard palate + medial nasal wall	IIIa	Double island RFFF + ICFF	None
4	6 × 5 × 5 cm	Maxillectomy + alveolar ridge + hard palate + medial nasal wall	IIIa	Double island RFFF + FFF	None
5	9 × 7 × 5 cm	Maxillectomy + alveolar ridge + zygomatic arch + medial nasal wall + orbit (total) + anterior cranial fossa+ ethmoidectomy (anterior, posterior)	IIIb	Double island bRFFF + VRAM	None
6	10 × 8 × 6 cm	Maxillectomy + alveolar ridge + hard palate + medial nasal wall + nasal septum + orbit + infratemporal fossa + palato-pterygoid fossa + medial pterygoid muscle + ethmoidectomy (anterior, posterior)	IIIb	Double island bRFFF + VRAM	Prolonged wound healing
7	6 × 5 × 4 cm	Maxillectomy + alveolar ridge + hard palate + medial nasal wall	IIIa	Double island RFFF + FFF	None
8	8 × 6 × 4 cm	Maxillectomy + alveolar ridge + hard palate + medial nasal wall	IIIa	Double island RFFF + FFF	None
9	8 × 4 × 5 cm	Maxillectomy + alveolar ridge + hard palate + medial nasal wall + buccal soft tissues	IIIa	Double island RFFF + FFF	None
10	9 × 6 × 3 cm	Maxillectomy + alveolar ridge + hard palate + medial nasal wall	IIIa	Double island RFFF + FFF	Ischemia of the radial flap skin
11	7 × 4 × 5 cm	Maxillectomy + alveolar ridge + hard palate + medial nasal wall + buccal soft tissues	IIIa	Double island RFFF + ICFF	Prolonged wound healing
12	7 × 6 × 4 cm	Maxillectomy + alveolar ridge + hard palate + medial nasal wall	IIIa	Double island RFFF + FFF	None
13	5 × 4 × 4 cm	Maxillectomy + alveolar ridge + hard palate + medial nasal wall	IIIa	Double island RFFF + ICFF	None
14	6 × 3 × 3 cm	Maxillectomy + alveolar ridge + hard palate + medial nasal wall	IIIa	Double island RFFF + FFF	None

**Table 4:** Raw UW-QOL domain scores presented for individual patients in the chimeric flap group. For functional domains (chewing and swallowing, speech, sensation, and social activity), lower raw scores indicate better function, whereas for symptom-oriented domains (pain and perceived change in appearance), lower raw scores indicate lower symptom impact.

No.	Type (Classification by Cordeiro)	Average Scores of the QOL Items					
		Pain (0–20) (a)	Chewing and Swallowing (0–8) (b)	Speech (0–8) (c)	Feeling (0–8) (d)	Change in Appearance (0–4) (e)	Social Activity (0–44) (f)
1	IIIa	2	3	3	1	0	42
2	IIIa	1	2	3	1	1	40
3	IIIa	1	1	0	0	0	40
4	IIIa	3	2	2	1	1	34
5	IIIb	4	2	1	2	2	32
6	IIIb	2	1	1	1	2	38
7	IIIa	1	2	1	2	1	40
8	IIIa	3	2	1	1	2	42
9	IIIa	4	3	2	3	2	32
10	IIIa	4	3	2	4	2	28
11	IIIa	1	2	1	1	1	36
12	IIIa	2	2	1	2	1	34
13	IIIa	2	1	0	1	0	42
14	IIIa	1	1	0	1	0	40

**Table 5:** Transformed (0–100) UW-QOL domain scores comparing chimeric and single free flap reconstruction at 6 months postoperatively. Values represent normalized domain-level scores and do not constitute a validated composite quality-of-life index.

Type of Reconstruction	Average transformed QOL scores					
	Pain	Chewing and Swallowing	Speech *	Sensation *	Change in Appearance *	Social Activity *
Chimeric free flaps	14.2	71.1	**84.4**	**87.7**	**69.3**	**73.6**
Single free flaps	17.5	60.4	**53.6**	**72.5**	**52.1**	**38.6**

* Exact *p*-values are reported in the Section 3. Statistical significance was defined as *p* < 0.05. *p*-values are unadjusted for multiple comparisons across domains.

## Data Availability

The original contributions presented in this study are included in the article. Further inquiries can be directed to the corresponding author.

## Supplementary Materials

The following supporting information can be downloaded at: https://www.mdpi.com/article/10.3390/jcm15051866/s1. Table S1: Raw-to-standardized QOL score conversion example.

## Abbreviations

The following abbreviations are used in this manuscript:

NCF	Natural Chimeric Flaps
PCF	Prefabricated Chimeric Flaps
ZMB	Zygomatico-Maxillary Bone
PMB	Pterygo-Maxillary Bone
NMB	Naso-Maxillary Buttresses
QOL	Quality Of Life
LD	Latissimus Dorsi Flap
FS	Function Scale
SS	Symptom Scale
