# Supplementary material for: Functional and Aesthetic Outcomes of Chimeric vs. Single Free Flaps in Midface Reconstruction Following Tumor Resection: A Retrospective Analysis
Source: J Clin Med. 2026 Feb 28;15(5):1866. doi: 10.3390/jcm15051866 (PMC12986017; doi:10.3390/jcm15051866)
Supplement: Supplementary file 1 [file jcm-15-01866-s001.zip › jcm-4156116-supplementary.pdf]

Journal of Clinical Medicine

MDPI

Supplementary Table S1. Raw-to-standardized QOL score conversion example

**UW-QOL Domain**	**Raw Score (RS)**	**Scale Range**	**Domain Type**	**Transformation Formula Applied**	**Standardized Score (0–100)**	**Interpretation**
Pain	3	0–20	Symptom	SS = (3–0)/(20–0)×100	15	Mild symptoms
Chewing / Swallowing	2	0–8	Functional	FS = (8–2)/(8–0)×100	75	Good function
Speech	1	0–8	Functional	FS = (8–1)/8×100	87.5	Near-normal speech
Sensation	2	0–8	Functional	FS = (8–2)/8×100	75	Moderate preservation
Appearance Change	1	0–4	Symptom	SS = (1–0)/(4–0)×100	25	Mild subjective change
Social Activity	38	0–44	Functional	FS = (44–38)/44×100	13.6	Limited restriction

 https://doi.org/10.3390/jcm15051866
